# Inattention, Impulsivity, and Hyperactivity among Individuals with Self-Reported Impaired Wound Healing

**DOI:** 10.3390/brainsci12080961

**Published:** 2022-07-22

**Authors:** Jessica Balikji, Maarten M. Hoogbergen, Johan Garssen, Joris C. Verster

**Affiliations:** 1 Division of Pharmacology, Utrecht Institute for Pharmaceutical Sciences, Utrecht University, 3584 CG Utrecht, The Netherlands; j.balikji@uu.nl (J.B.); j.garssen@uu.nl (J.G.); 2 Division of Plastic Surgery, Catharina Ziekenhuis, Michelangelolaan 2, 5623 EJ Eindhoven, The Netherlands; dr.mmhoog@gmail.com; 3 Global Centre of Excellence Immunology, Nutricia Danone Research, 3584 CT Utrecht, The Netherlands; 4 Centre for Human Psychopharmacology, Swinburne University, Melbourne, VIC 3122, Australia

**Keywords:** attention deficit, hyperactivity, impulsivity, impaired wound healing, slow healing wounds, wound infection, ADHD, perceived immune fitness

## Abstract

Background: Inattention and impulsivity are common causes of accidents and injury. The aim of the current study was to examine the level of attention deficit (AD), hyperactivity, and impulsivity (HI) in individuals with and without self-reported impaired wound healing (IWH). Methods: A survey was conducted among N = 773 Dutch young adults, 18–30 years old. N = 198 were allocated to the IWH group and N = 575 to the control group. All participants completed the Attention Deficit Hyperactivity Disorder (ADHD) Rating Scale. Results: The analysis revealed that the IWH group has significantly higher scores on AD and HI, compared to the control group. Among the IWH group, 12.8% screened positive for AD (compared to 5.8% of the control group) and 14.0% screened positive for HI (compared to 7.4% of the control group). Conclusion: Clinically relevant increased inattention, impulsivity, and hyperactivity were observed among individuals with self-reported impaired wound healing.

## 1. Introduction

There is overwhelming evidence that both inattention and increased impulsivity and hyperactivity levels are associated with having an increased risk of accidents and injury [1,2,3,4,5,6,7,8]. This is particularly evident in clinical populations that are characterized by attention deficit (AD) and hyperactivity, impulsivity (HI), such as patients with attention deficit hyperactivity disorder (ADHD). For example, in adults with ADHD, impaired on-road and simulated driving [9,10,11], increased numbers of accidents and injury [12,13], and increased driver penalties (e.g., speeding tickets) have been consistently reported and associated with the core deficits of ADHD [14,15,16].

It can be hypothesized that other psychological and/or medical conditions for which patients report AD or HI may also make these patients more vulnerable for having accidents and injury. Several psychological and medical conditions have ADHD as comorbidity, including mood disorders (e.g., anxiety, depression), substance use disorders, personality disorders, autism, and insomnia [17]. In addition, neurological and digestive diseases have been associated with ADHD symptomatology as well [18,19,20]. As such, it has been suggested that the gut–brain axis may play a common role in in the pathology of both ADHD and these co-occurring diseases [21,22,23,24,25,26]. Moreover, in wound healing, the gut–brain axis plays an important role [27,28], with adequate immune functioning as an important determinant of adequate wound healing [29]. Based on the shared involvement of the gut–brain axis and the immune system, it could be hypothesized that AD and HI are more frequently experienced in patients with immune-related diseases. Indeed, comorbid ADHD has been reported for asthma, allergic rhinitis, atopic eczema [19,20], obesity and being overweight [30], and patients with altered gut microbiome functions [14]. It would be worrisome if levels of AD and HI were increased in patients who suffer from impaired wound healing, i.e., patients with slow healing wounds and/or wound infection, as for these patients the consequences of accidents and injury are much more severe than for individuals with normal wound healing.

Although there is no literature on accident and injury risk of patients with impaired wound healing, there is indirect evidence that may support the hypothesis that AD may be common among patients with impaired wound healing. Ongoing treatment (e.g., daily wound management) can be a significant burden for patients with chronic wounds. Wound-related symptoms, such as pain and physical discomfort, may have a significant negative impact on quality of life [31,32,33] and mood [33,34,35,36,37], and they can result in feelings of loss of self-control [38]. However, of more importance, the impaired wound healing may occupy the patient’s thoughts and distract patients from paying attention to potentially dangerous activities, such as driving a car.

The aim of the current study was to examine the level of AD and HI in individuals with and without self-reported impaired wound healing. General literature on negative mood and pain consistently show that these conditions are often accompanied by concentration problems and reduced alertness [39]. It is, therefore, reasonable to assume that the negative mood and pain that accompanies having chronic wounds, may also elicit AD among these patients and distract their attention in risky situations. It was, therefore, hypothesized that AD levels among individuals with IWH will be greater than the control group. No literature has been published on HI in relation to impaired wound healing. Therefore, no a priori hypothesis was formulated with regard to possible differences in HI between individuals with and without self-reported impaired wound healing.

## 2. Materials and Methods

Data from Otten et al. [40] was re-evaluated. For this study, a convenience sample of students from Utrecht University, the Netherlands, 18 to 30 years old, was recruited on university campus to complete a paper-and-pencil survey. The study was conducted in 2016 and informed consent was obtained from all participants. No ethics approval was required for this study according to the Central Committee of Research Involving Human Subjects, the Netherlands.

Subjects indicated whether or not they had experienced wound infection and/or slow healing wounds during the past year. Using the outcome of these two questions, subjects were allocated to (1) a control group that answered ‘no’ to both questions, (2) an IWH group that reported experiencing wound infection and/or slow healing wounds.

The ADHD Rating Scale (ARS) was completed to assess AD and HI symptoms [41]. The scale consists of 23 items that can be rated on a 4-point scale (0 = rarely or never, 1 = sometimes, 2 = often, 3 = very often). An overall ADHD rating was obtained by calculating the sum score of the 23 items. In addition, AD and HI were assessed with two subscales. There are different criteria for subjects to screen positive for having AD or HI. In the literature, cut-off scores for AD and HI of ≥5 (based on DSM-5 [42]) and ≥4 (based on Kooij et al., 2008 [41]) are currently used. To provide more insight in the data for each group, percentages of individuals that screen positive for AD or HI were computed. This was done for each cutoff score in the range of ≥0 to ≥10. Finally, the survey included a question about whether subjects were formally diagnosed for having ADHD.

Perceived immune fitness was assessed with a 1-item scale ranging from very poor (0) to excellent (10) [43]. The test–retest reliability of the 1-item perceived immune fitness score is 0.887 [44]. The subjects were further asked whether they experienced reduced immune fitness at the moment of completion of the survey (yes/no question).

Statistical analyses were conducted with SPSS (IBM Corp. Released 2013. IBM SPSS Statistics for Windows, Version 28.0. Armonk, NY, USA: IBM Corp.). Comparisons between the IWH and control groups were conducted with the Independent-Samples Mann–Whitney U Test. For data expressed as percentages, the IWH group and control group were compared with the “N-1” Chi-squared test, using a comparison of proportions calculator (MedCalc Software Ltd., Ostend, Belgium), available at https://www.medcalc.org/calc/comparison_of_proportions.php (Version 20.106; accessed on 13 May 2022). Differences between the groups were considered statistically significant if *p* < 0.05.

## 3. Results

N = 773 subjects (62.1% women) completed the survey. Demographics and perceived immune fitness of the subjects are listed in Table 1.

Compared to the control group, the IHW group comprised significantly more women, and was slightly but statistically significant younger. No other significant differences in demographics were observed between the two groups. The IWH group reported significantly lower perceived immune fitness and a significantly higher percentage of reduced immune fitness compared to the control group. AD and HI outcomes for the IWH and control group are summarized in Table 2.

**Figure 1:**
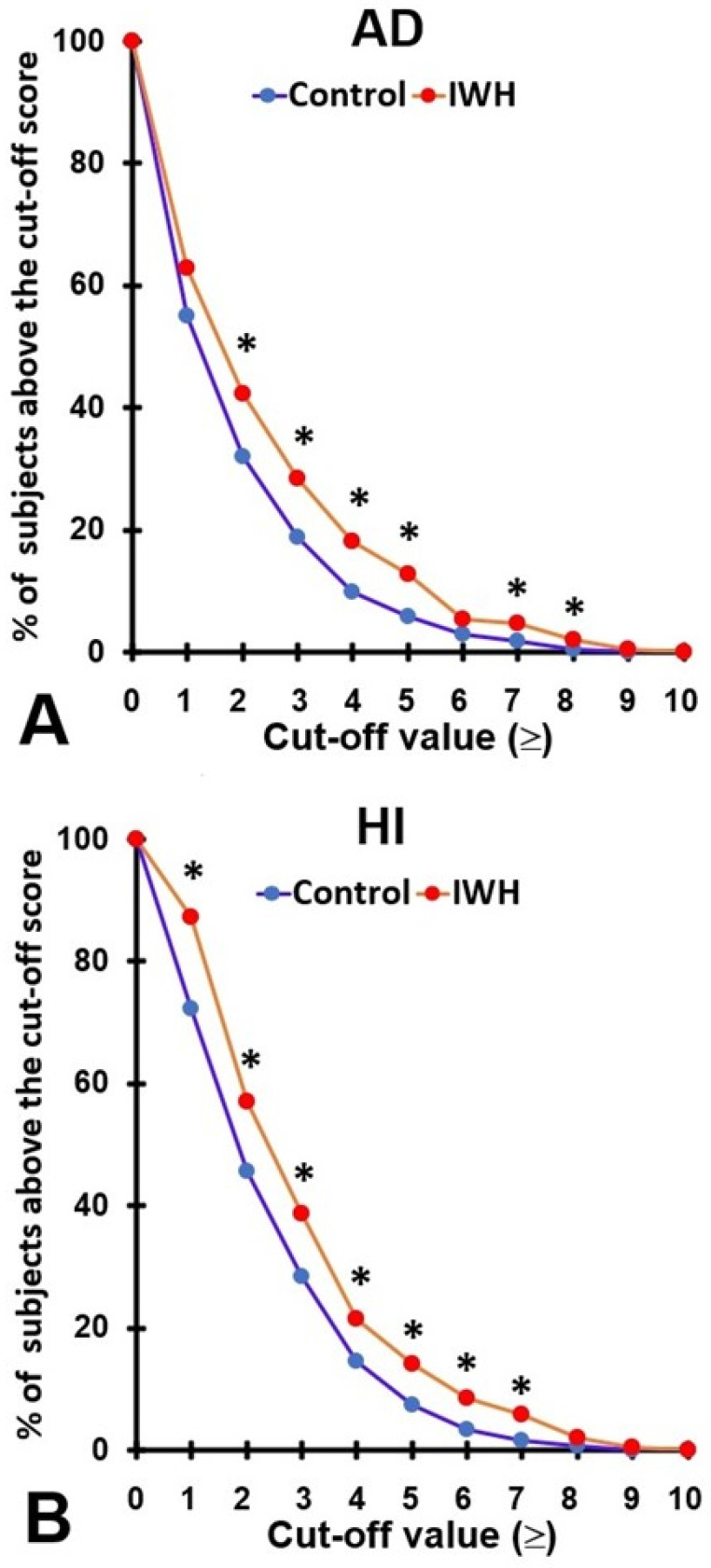
Percentage of subjects scoring positive for AD or HI, according to different cut-off values. (**A**) shows the results for attention deficit, (**B**) shows the results for hyperactivity, impulsivity. Significant differences between the IWH group and the control group (*p* < 0.05) are indicated by *. Abbreviations: AD = attention deficit, HI = hyperactivity, impulsivity.

Compared to the control group, the overall ADHD Rating Scale scores and those of the attention deficit and hyperactivity, impulsivity subscales were significantly higher in the IWH group (See Table 2). Figure 1 shows the percentages of subjects that score positive for AD (Figure 1a) or HI (Figure 1b), according to different cut-off values. Significant higher percentages were reported for the IWH group across all cut-off points and the observed differences were almost always statistically significant. Most notably are the cut-off values ≥ 4, proposed by Kooij et al. [41], and ≥ 5, in accordance with the DSM-5 [42]. For these cut-off values, the percentage of individuals that score positive for AD or HI were also significantly higher in the IWH group (See Table 2 and Figure 1).

## 4. Discussion

The analyses revealed that ratings of both AD and HI were significantly greater among individuals with self-reported impaired wound healing. In particular, the percentages of individuals that screened positive for AD and HI were much larger in the IWH group than the control group. The IWH group reported a positive screen for 12.8% (compared to 5.8% in the control group) and 14.0% for HI (compared to 7.4% in the control group). These statistically significant and clinically relevant observations are in line with the hypothesis that the discomfort and pain associated with having chronic wounds distracts attention from planned activities and social interactions [38]. However, as to date there is no comparative published data on AD and HI in patients with impaired wound healing, more research is warranted. There is an overwhelming amount of literature that demonstrated that inattention, impulsivity, and hyperactivity are associated with an increased risk of having accidents of injury [45]. In this context, the observation that a significantly greater percentage of individuals with impaired wound healing screen positive for AD and HI is worrisome. Especially in individuals with impaired wound healing it is essential to prevent having accidents and injury, as the potential wounds due to injury are more likely to require long-term treatment. Over the years, there have been changes in the selected cut-off point to screen positive for AD and HI. Therefore, in the current study these percentages were calculated for these different cutoff points (see Figure 1). The results show that across the range of possible cutoff points, the percentage of positive screens among the IWH group are consistently and significantly higher compared to the control group.

The observed effects on attention, hyperactivity, and impulsivity may be explained by prefrontal cortex dysfunctioning and associated altered inhibitory control, which characterizes patients with ADHD [46]. However, research on brain functioning or inhibitory control of individuals with IWH is currently lacking. Therefore, future research should evaluate brain functioning of individuals with IWH to evaluate possible explanations for the association between AD/HI and IWH by considering neurobiological and immunological factors that characterize the individual with ADHD.

In order to interpret the current data correctly, several limitations of the study should be considered. Firstly, the data were collected retrospectively. As such, recall bias may have influenced the study outcomes. Prospective studies with real-time assessments should be done to confirm our findings. Secondly, participants were allocated to the impaired wound healing group or control group. It is important to note that this study was based on self-reported data and no formal diagnosis was obtained to verify this. It is recommended that future studies should confirm wound healing status by diagnosis made by a clinician. Thirdly, the study comprised a convenience sample of Dutch students aged 18 to 30 years old. Therefore, it is unclear to what extent our findings can be generalized to other age groups or extrapolated to the general population. Fourthly, no information was collected about the possible underlying diseases or other relevant health characteristics of the participants of this study that could aid the interpretation of the study outcomes. Finally, the presented correlations do not imply causality, and directional conclusions cannot be drawn from the data.

Notwithstanding these limitations, participants with self-reported impaired wound healing reported significantly higher scores of AD and HI, and significantly greater percentages of positive screens for AD and HI were found for the IWH group. These findings justify further research on this topic.

## 5. Conclusions

Significant higher ratings of impulsivity and/or hyperactivity and attention deficits were reported by individuals with self-reported impaired wound healing. Given its potential consequences in terms of having accidents or injury, and thus acquiring chronic wounds, these findings justify further research.

## Figures and Tables

**Table 1:** Demographics.

	Control Group (N = 575)	IWH Group (N = 198)	*p*-Value
Sex (m/f) (%)	40.7/59.3	29.7/70.3	0.008 *
Age	21.6 (2.7)	21.0 (2.4)	0.008 *
BMI (kg/m^2^)	22.2 (2.9)	22.2 (2.6)	0.897
Alcohol consumption (% yes)	81.6%	82.2%	0.693
Number of drinks per week ^1^	7.3 (8.7)	8.7 (12.4)	0.079
Smoking (% yes)	12.6%	12.6%	0.233
Perceived immune fitness	7.8 (1.2)	7.5 (1.2)	<0.001 *
Reduced immune fitness (%)	21.9%	34.9%	0.002 *
Diagnosed ADHD (%)	3.0%	4.5%	0.359

Significant differences with the IWH and control group (*p* < 0.05) are indicated by ^1^: Mean (SD) for the subsample of subjects that consume alcohol. *. Abbreviations: IWH = impaired wound healing, BMI = body mass index, ADHD = attention deficit hyperactivity disorder.

**Table 2:** ADHD outcomes.

	Control Group	IWH Group	*p*-Value
ADHD Rating Scale overall score	14.2 (6.8)	16.8 (7.4)	<0.001 *
Attention deficit subscale	1.3 (1.6)	1.8 (2.1)	0.001 *
AD% ≥ 4, Kooij et al. [41]	9.9%	18.2%	0.003 *
AD% ≥ 5, DSM-5 [42]	5.8%	12.8%	0.002 *
Hyperactivity, impulsivity subscale	1.7 (1.7)	2.4 (2.0)	<0.001 *
HI% ≥ 4, Kooij et al. [41]	14.6%	21.5%	0.028 *
HI% ≥ 5, DSM-5 [42]	7.4%	14.0%	0.007 *

Significant differences between the IWH and control group (*p* < 0.05) are indicated by *. Abbreviations: ADHD = attention deficit hyperactivity disorder, AD = attention deficit, HI = hyperactivity, impulsivity, IWH = impaired wound healing.

## Data Availability

The data is available from the corresponding author upon reasonable request.
